# Plasma microRNA and metabolic changes associated with pediatric acute respiratory distress syndrome: a prospective cohort study

**DOI:** 10.1038/s41598-022-15476-0

**Published:** 2022-08-26

**Authors:** Denis J. Ohlstrom, Christina Sul, Christine U. Vohwinkel, Laura Hernandez-Lagunas, Anis Karimpour-Fard, Peter M. Mourani, Todd C. Carpenter, Eva S. Nozik, Carmen C. Sucharov

**Affiliations:** 1 Developmental Lung Biology and Cardiovascular Pulmonary Research Laboratories, Departments of Pediatrics and Medicine, University of Colorado, Anschutz Medical Campus, Aurora, CO USA; 2 Department of Pharmacology, University of Colorado, Anschutz Medical Campus, Aurora, CO USA; 3 Division of Pediatric Critical Care, Department of Pediatrics, University of Colorado, Anschutz Medical Campus, Aurora, CO USA; 4 Section of Pediatric Critical Care, Department of Pediatrics, University of Arkansas for Medical Sciences, Arkansas Children’s Research Institute, Little Rock, AR USA; 5 Division of Cardiology, Department of Medicine, University of Colorado, Anschutz Medical Campus, 12700 E 19th Ave B139, Aurora, CO 80045 USA

**Keywords:** Biomarkers, Molecular medicine

## Abstract

Acute respiratory distress syndrome is a heterogeneous pathophysiological process responsible for significant morbidity and mortality in pediatric intensive care patients. Diagnosis is defined by clinical characteristics that identify the syndrome after development. Subphenotyping patients at risk of progression to ARDS could provide the opportunity for therapeutic intervention. microRNAs, non-coding RNAs stable in circulation, are a promising biomarker candidate. We conducted a single-center prospective cohort study to evaluate random forest classification of microarray-quantified circulating microRNAs in critically ill pediatric patients. We additionally selected a sub-cohort for parallel metabolomics profiling as a pilot study for concurrent use of miRNAs and metabolites as circulating biomarkers. In 35 patients (n = 21 acute respiratory distress, n = 14 control) 15 microRNAs were differentially expressed. Unsupervised random forest classification accurately grouped ARDS and control patients with an area under the curve of 0.762, which was improved to 0.839 when subset to only patients with bacterial infection. Nine metabolites were differentially abundant between acute respiratory distress and control patients (n = 4, both groups) and abundance was highly correlated with miRNA expression. Random forest classification of microRNAs differentiated critically ill pediatric patients who developed acute respiratory distress relative to those who do not. The differential expression of microRNAs and metabolites provides a strong foundation for further work to validate their use as a prognostic biomarker.

## Introduction

Acute respiratory distress syndrome (ARDS) is a complex, rapidly progressing, and often fatal condition affecting about 10% of critically ill adults, with an estimated mortality ranging from 30 to 40%^1^. While the incidence of ARDS in pediatric populations is lower than in adults, comprising 1–3% of all Pediatric ICU admissions, the mortality of pediatric ARDS (PARDS) remains high at 17–33%^2,3^. ARDS has a complex pathophysiology resulting from diverse etiologies such as bacterial and viral lung infections, sepsis, and trauma. ARDS remains defined by clinical criteria which do not account for this heterogeneity. Current definitions of both adult and pediatric ARDS focus on clinical features such as chest radiograph findings, oxygenation, and mean airway pressure parameters^4^. However, emerging evidence suggests there is considerable biological and genetic heterogeneity of the ARDS disease process, with distinct subphenotypes of patients that have different disease risk and response to therapies. This new information drives the need for a more personalized approach to diagnosis and management^5^.

A targeted approach to subphenotyping ARDS will require improved biomarkers to identify patients at risk of poor outcomes. microRNAs (miRNAs) are small, single-stranded, noncoding RNAs that regulate a wide array of cellular processes via mRNA degradation or translational repression^6^. Next-generation sequencing studies in humans and animal models have uncovered a large number of miRNAs dysregulated with ARDS^7^. In preclinical studies, our group and others have shown that miRNA levels can influence disease development and progression and may be a therapeutic target^8,9^. miRNAs are stable in the circulation and have emerged as promising biomarkers in a variety of pathologies^10^. In addition to miRNAs, other relevant biomarkers, such as circulating metabolites, may be useful in stratifying risk for ARDS^11^. Recent advances in metabolomics have enabled the evaluation of circulating metabolites in a variety of disease processes including ARDS^12^.

Our broad goal is to determine if circulating miRNAs and metabolites may serve as biomarkers to stratify risk of developing PARDS. As a first step, we conducted a single-center prospective cohort study to test the hypothesis that circulating miRNA populations can be a diagnostic biomarker of ARDS and that changes in circulating miRNAs are associated with changes in circulating metabolites.

## Materials and methods

### Patient sample collection

This prospective observational study was performed in the Pediatric Intensive Care Unit (PICU) at Children’s Hospital Colorado as part of a larger study of airway microbiome changes in relation to ventilator-associated pneumonia^13^. The study was approved by the Colorado Multiple Institutional Review Board (COMIRB #14-1530), and informed consent was obtained from the child’s parents or legal guardian. All methods were performed according to relevant guidelines and regulations. Eligible study subjects were 31 days to 18 years of age and were expected by their primary clinical team to be intubated ≥ 72 h at enrollment. Exclusion criteria specific to this study were known chromosomal abnormalities, cancer not in remission, history of stem cell transplantation, blood product administration, plasma exchange therapies, or dialysis within 7 days. Patients in the ARDS group met criteria for moderate to severe ARDS using modified Berlin Criteria adjusted for altitude at our site, defined as acute onset (< 7 days from triggering event), bilateral infiltrates on chest radiograph, and PaO_2_ to FiO_2_ (P/F) ratio < 180 or SaO_2_ to FiO_2_ (S/F) ratio of < 230 on at least 3 time points each separated by at least 1 h in a 24-h period^14^. Patients included in the control group were critically ill and mechanically ventilated but did not meet criteria for ARDS during their PICU stay. Enrolled patients were tested as clinically indicated for respiratory viruses and endotracheal bacteria. All 21 ARDS patients and 9 of 14 controls were tested for both viruses and bacteria prior to sample collection; control patients not tested were considered negative. As a measure of severity of illness, Pediatric Logistic Organ Dysfunction (PELOD) scores were calculated for the day of sample collection^15^. Plasma samples for miRNA analysis were collected in EDTA tubes, centrifuged, aliquoted, and stored at − 80 °C within 30 min of collection. All 21 ARDS patients and 12 of 14 control patients were endotracheally intubated on PICU day 1 or 2. One control patient was intubated on PICU day 3, and 1 additional control patient was intubated on PICU day 4. ARDS criteria were met on day 1 or 2 of intubation for 19 of 21 ARDS patients; 1 patient developed ARDS on day 3 of intubation and 1 patient developed ARDS on day 4 of intubation. Plasma samples were collected between days 1 and 4 of moderate to severe ARDS for all patients, with 19 of 21 samples collected within 3 days of ARDS onset. Samples were collected between days 1 and 5 of endotracheal intubation for all patients.

### miRNA array

miRNA arrays were performed as described previously^16^. Briefly, 3 µl of plasma from each subject was submitted to three cycles of heat/freeze to ensure miRNAs are released from microvesicles or interactions with proteins. miRNAs were reverse transcribed using a pool of primers specific for each miRNA (ThermoFisher Scientific). Real-time PCR reactions were performed in a 384 well TaqMan Low Density Array (ThermoFisher Scientific) containing sequence-specific primers and TaqMan probes in the ABI 7900HT.

### Array analysis

Raw Ct values were normalized to miR-320 based on our unpublished results showing expression of circulating miR-320 is the least variable in over 400 pediatric samples (not shown). Statistical significance was tested using Wilcoxon sign-rank test. Random forest classification (RFC) was executed in R using 50,000 trees to identify the top 3 miRNAs that differentiated comparison groups (https://cran.r-project.org/web/packages/randomForest/randomForest.pdf). The RFC model’s sensitivity and specificity were assessed by receiver operating curve (ROC) using the top three differentiating miRNAs. Heatmap was plotted using the heatmap.2 function in ggplot2 package in R^17^. Area under the receiver operating characteristic curve (AUC) was calculated using the pROC package^18^. Circos plot was generated using the circacompare package^19^.

### Pathway analysis

Predicted pathway analysis based on altered miRNAs was done using the miRNA Enrichment Analysis and Annotation Tool (miEAA)^20^. All 15 dysregulated miRNAs were evaluated. Pathways with a qvalue of < 0.003 or pathways associated with metabolite-miRNA pairs are shown.

### Metabolomic sample extraction and quantification

Plasma samples (10 µl) were extracted with 240 µl of ice cold methanol:acetonitrile:water (5:3:2) via vigorous vortexing for 30 min at 4 C. Supernatants were clarified by centrifugation (10 min, 18,213 rcf, 4 °C) and analyzed on a Thermo Vanquish ultra-high pressure liquid chromatograph coupled online to a Thermo Q Exactive mass spectrometer. Metabolites were separated using a 5 min C18 gradient method in positive and negative modes exactly as described previously (separate runs, 20 µL per injection)^21^. Peak integration and metabolite assignment were performed using Maven (Princeton University) against the KEGG database, confirmed against chemical formula determination from isotopic patterns and accurate mass, and validated against experimental retention times for > 650 standard compounds (Sigma Aldrich; MLSMS, IROATech, Bolton, MA, USA)^22^. Instrument stability was assessed using replicate injections of a quality control mix injected every 4 runs.

### Metabolomics and miRNA-metabolite integrated analysis

Plasma samples (10 µl) were extracted with 240 µl of ice cold methanol:acetonitrile:water (5:3:2) as described previously^23^. Differentially abundant metabolites were identified by absolute fold change > 1.5 and *p* < 0.05 by *t*-test (n = 4). Differentially expressed miRNAs from matched samples were identified using a *t*-test (*p* < 0.05). Significantly different miRNAs and metabolites were correlated using Pearson correlation in R.

### Ethical approval

The study was approved by the Colorado Multiple Institutional Review Board (COMIRB #14–1530), and informed consent was obtained from the child’s parents or legal guardian.

## Results

### Patient characteristics

The study cohort included 21 ARDS patients (11 moderate and 10 severe) and 14 control patients. Demographic characteristics and clinical characteristics are shown in Table 1. As compared to controls, ARDS patients tended to be younger, with a significant increase in lower respiratory tract infections (LRTI, 76% vs. 21%, *p* = 0.002), and significantly more viral respiratory infections (90% vs. 29%, *p* < 0.001). Viral LRTI was identified in 23 patients including primary and non-primary diagnoses (90% ARDS, 29% control, *p* < 0.001); 13 of those patients had both viral and bacterial pathogens identified (10 ARDS, 3 control), and 10 patients had only viral infection (9 ARDS, 1 control). Bacterial LTRI was diagnosed in 19 patients (11 ARDS, 8 control), and 6 of those patients had only bacterial infection (1 ARDS, 5 control). Neither viral nor bacterial infection was documented in 6 patients (1 ARDS, 5 control). PELOD-2 scores, PICU length of stay, ventilator-free days, days of non-invasive ventilation following extubation, and days on oxygen therapy prior to hospital discharge were similar between ARDS and control groups.

**Table 1:** Demographics and clinical characteristics. ARDS-acute respiratory distress syndrome, LRTI-lower respiratory tract infection, TBI-traumatic brain injury, PELOD-pediatric logistic organ dysfunction, NIV-non-invasive ventilation, PICU-pediatric intensive care unit.

Characteristic	Control	ARDS	*p*-value
	(n = 14)	(n = 21)	
Age (median, yrs)	3.9 (IQR = 0.5–10.7)	1.4 (IQR = 0.7–2.2)	0.35
Sex (% male)	71%	67%	0.81
Primary diagnoses (n, %)			
LRTI	3 (21%)	16 (76%)	0.002
Sepsis	4 (29%)	2 (10%)	0.15
TBI	4 (29%)	0	0.01
Other	3 (21%)	3 (15%)	0.65
Viral respiratory infection (n, %)	4 (29%)	19 (90%)	< 0.001
Bacterial respiratory infection (n, %)	8 (57%)	11 (52%)	0.77
PELOD (mean ± SD)	7.0 ± 1.5	6.7 ± 2.0	0.34
Ventilator-free days (mean ± SD)	21.3 ± 6.5	20.8 ± 5.6	0.49
NIV days post-extubation (mean ± SD)	0.9 ± 1.5	1.4 ± 1.8	0.38
Days of oxygen therapy (mean ± SD)	9.3 ± 9.9	15.0 ± 25.6	0.34
PICU length of stay (mean ± SD)	9.6 ± 9.3	14.9 ± 25.4	0.33

### Unsupervised RFC and unsupervised hierarchical clustering of ARDS versus control patients

**Figure 1:**
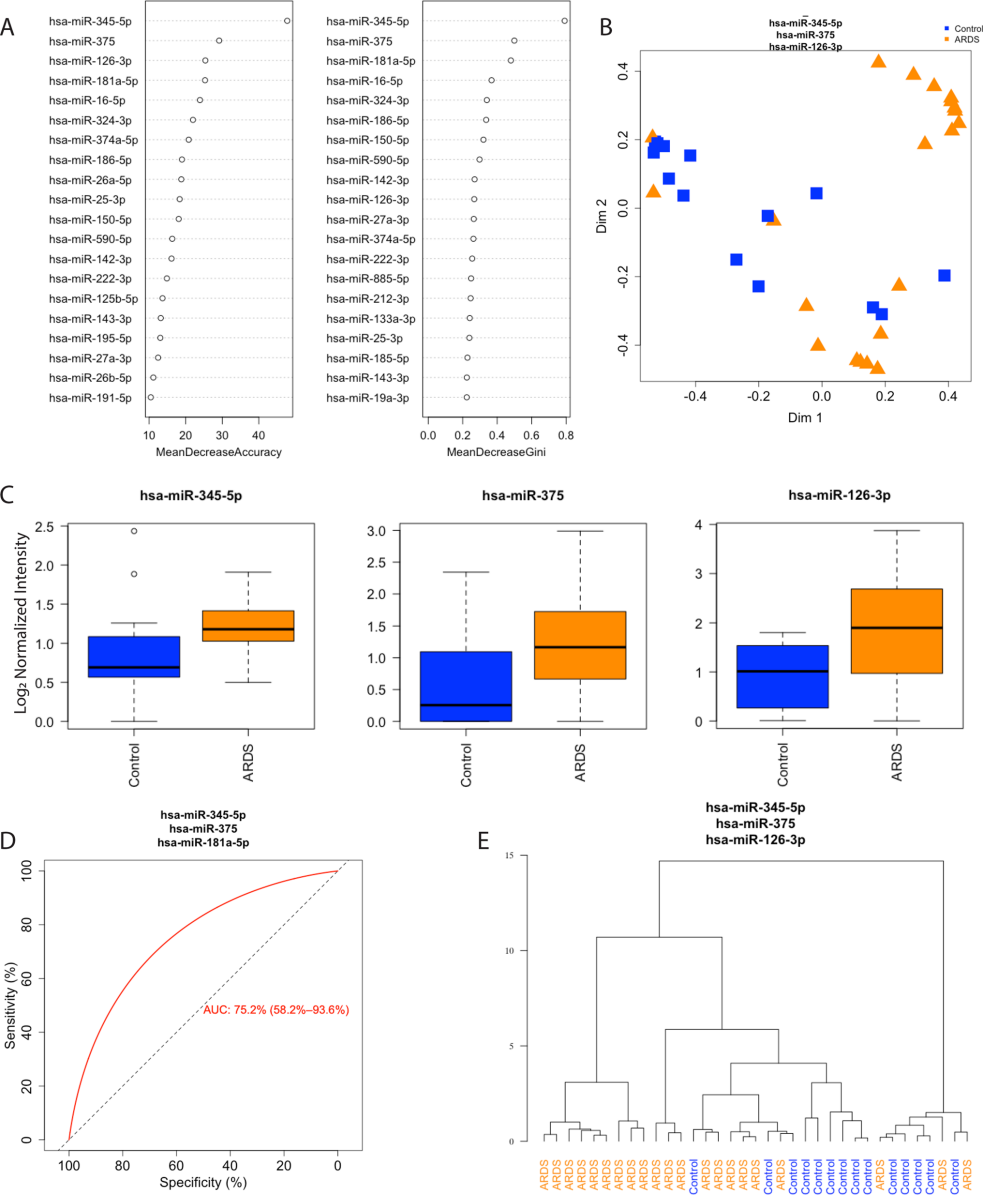
Random forest classification (RFC) of acute respiratory distress syndrome (ARDS) and control patients (n = 21 ARDS, n = 14 control). (**A**) Ranked miRNA importance in patient identification by unsupervised RFC of differentially expressed miRNAs. (**B**) RFC of ARDS and control patients using the top 3 miRNAs. (**C**) Mean relative quantification of the top three miRNAs identified by RFC, black line for the median and whiskers for the 25th and 75th percentiles. (**D**) Receiver-operating curve for classification of samples based on the top three miRNAs identified by RFC. (**E**) Unsupervised hierarchical clustering using the three miRNAs identified by RFC. ARDS-Acute respiratory distress syndrome, miRNA-microRNA.

Fifteen miRNAs were differentially expressed between ARDS and control patients, all of which were higher in ARDS patients (Table S1, Fig. S1). Unsupervised RFC identified miRNA-345-5p, -375-3p, and -126-3p as the best discriminators of ARDS from control patients as demonstrated by the highest mean decrease in accuracy and Gini coefficient when removed from the classifier (Fig. 1A,B). All three miRNAs were higher in ARDS patients; miRNA-345-5p, -375-3p, and -324-3p increased 1.29, 1.62 and 1.78 fold respectively (Fig. 1C, Table S1). Hierarchical clustering distinguished ARDS patients from controls (Fig. 1D). RFC of miRNA profiles identified disease status with an area under the curve of 0.762 (Fig. 1E).

### Unsupervised RFC and hierarchical clustering ARDS versus control in bacterially infected patients

**Figure 2:**
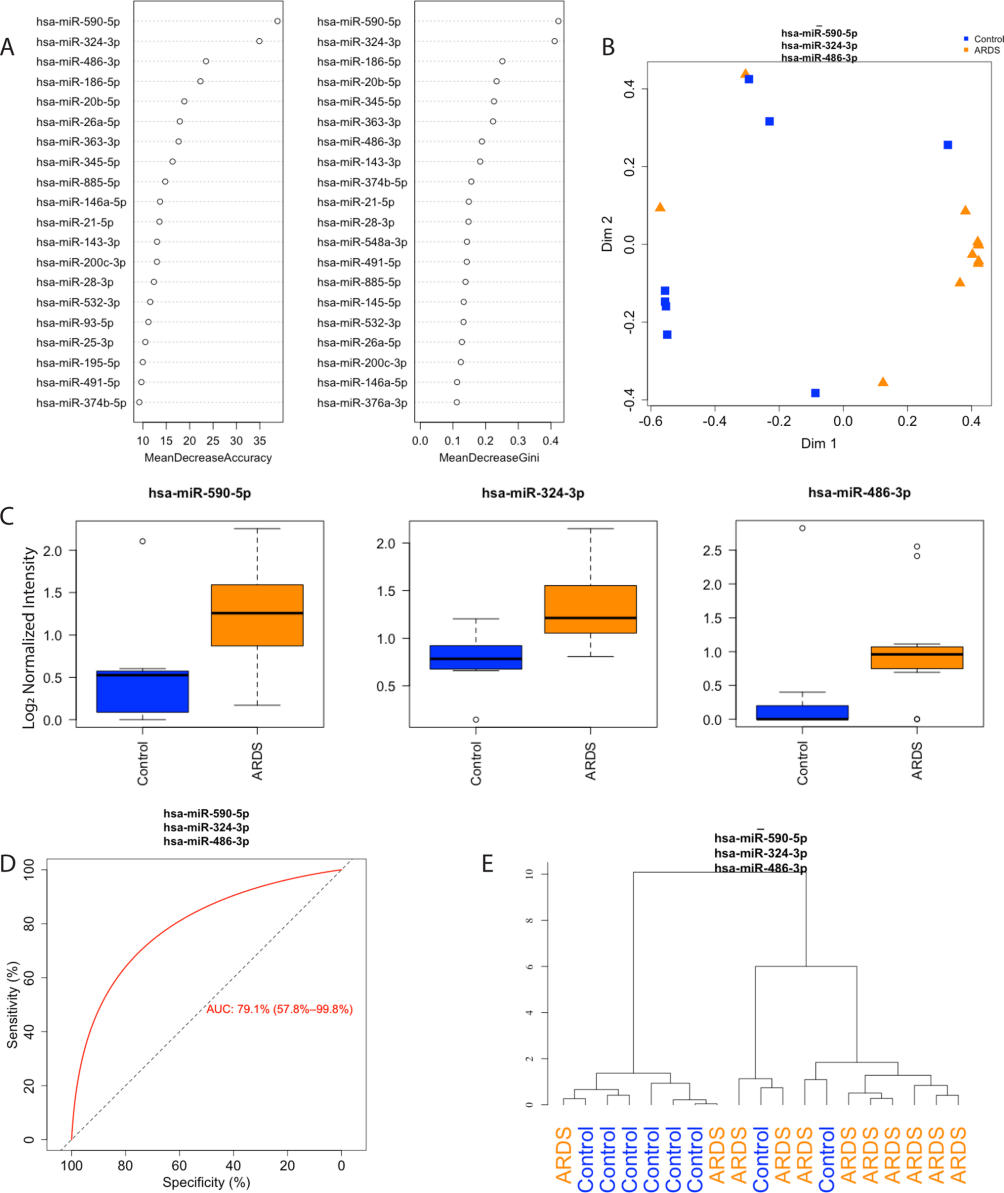
Random forest classification (RFC) of bacterially infected acute respiratory distress syndrome (ARDS) and control patients (n = 11 ARDS, n = 8 control). (**A**) Ranked miRNA importance in patient identification by unsupervised RFC of differentially expressed miRNAs. (**B**) RFC of bacterially-infected ARDS and control patients using the top 3 miRNAs. (**C**) Relative quantification of the top three miRNAs identified by RFC, black line for the median and whiskers for the, 25th and 75th percentiles. (**D**) Receiver-operating curve for classification of samples based on the top three miRNAs identified by RFC. (**E**) Unsupervised hierarchical clustering using the three miRNAs identified by RFC. ARDS-Acute respiratory distress syndrome, miRNA-microRNA.

We next sought to determine if limiting our analysis to patients with documented bacterial lower respiratory tract infections would improve the performance of circulating miRNAs (n = 11 ARDS, n = 9 control). Unsupervised RFC identified miRNA-590-5p, -324-3p, and -486-3p as the top three miRNAs capable of differentiating ARDS from controls (Fig. 2A) as shown by the highest in mean decrease in accuracy and gini coefficient (Fig. 2B). miRNA-590-5p, -324-3p, and -486-3p were 1.59, 1.47 and 1.55 fold higher in ARDS patients respectively (Fig. 2C, Table S2). Unsupervised hierarchical clustering using the top three miRNAs show these miRNAs can distinguish ARDS patients from controls (Fig. 2D). RFC of miRNA profiles identified disease status with an AUC of 0.839 (Fig. 2E).

### Unsupervised RFC and hierarchical clustering of ARDS patients with viral versus bacterial and viral infection

**Figure 3:**
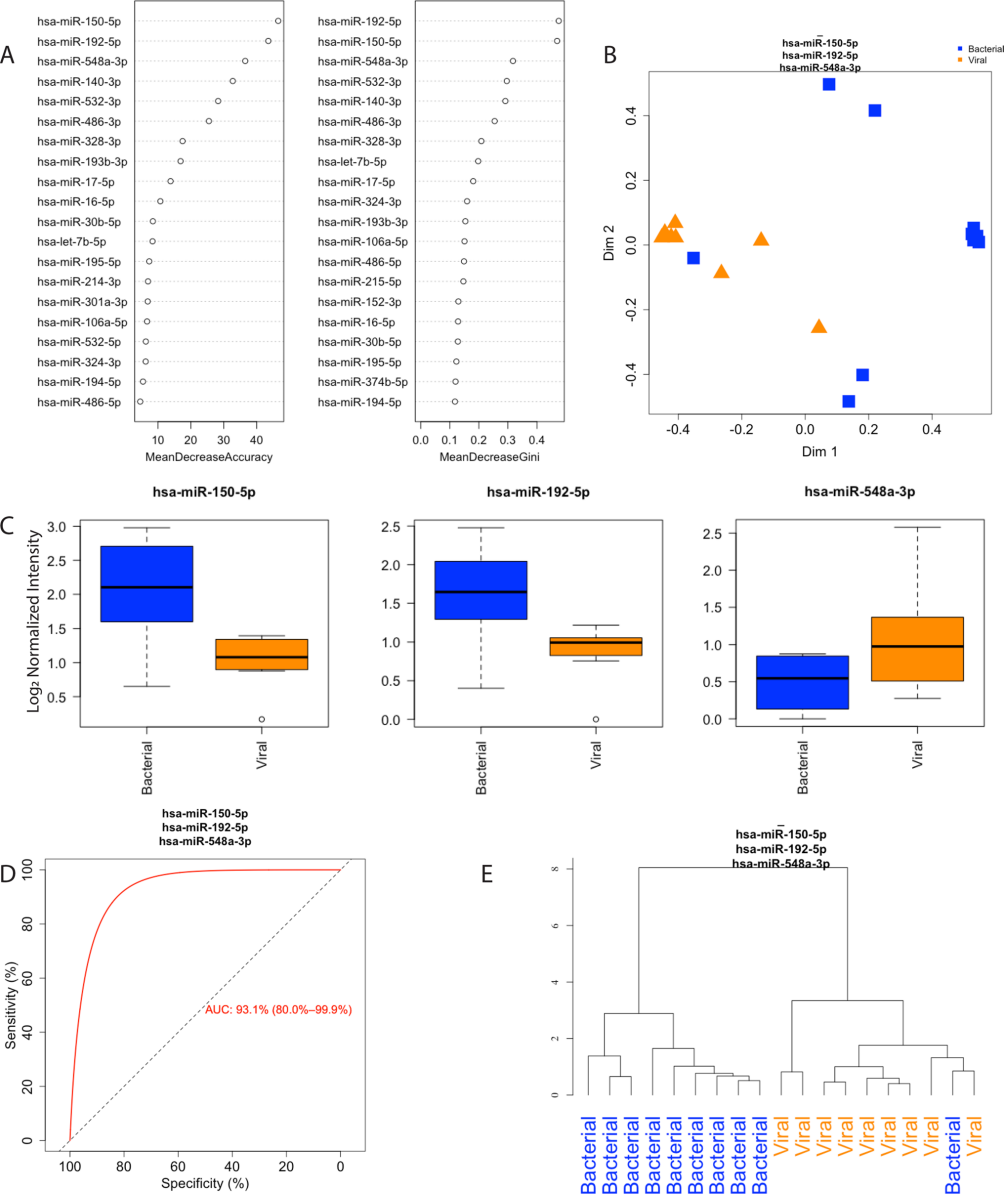
Random forest classification (RFC) of virally vs bacterially infected acute respiratory distress syndrome (ARDS) patients (n = 10 bacterial, n = 9 viral). (**A**) Ranked miRNA importance in patient identification by unsupervised random forest classification (RFC) of differentially expressed miRNAs. (**B**) RFC of patients with viral vs bacterial infection using the top 3 miRNAs. (**C**) Relative quantification of the top three miRNAs identified by RFC, black line for the median and whiskers for the 25th and 75th percentiles. (**D**) Receiver-operating curve for classification of samples based on the top three miRNAs identified by RFC. (**E**) Unsupervised hierarchical clustering using the three miRNAs identified by RFC. ARDS-Acute respiratory distress syndrome, miR-microRNA.

To determine if circulating miRNAs can differentiate infectious etiology in ARDS, 9 ARDS patients with only viral infection and 10 patients with bacterial and viral infections were compared using unsupervised RFC. Random forest classification using the top three miRNAs distinguished bacterially infected and virally infected patients (Fig. 3A). Unsupervised RFC based on mean decrease in accuracy and gini coefficient are shown in Fig. 3B. miRNAs-150-5p and -192-5p increased 1.98 and 1.68 fold respectively, while miRNA-548a-3p decreased -1.54 fold (Fig. 3C, Table S3). Unsupervised hierarchical clustering using the top three miRNAs showed these miRNAs can group ARDS patients according to infection type (Fig. 3D). RFC yielded an AUC of 0.894 to differentiate viral from bacterial/viral-infected ARDS patients (Fig. 3E).

### Pathway analysis of altered miRNAs

To define the possible biological significance of altered miRNAs, we used miEAA to evaluate predicted KEGG pathways^24^ of miRNAs altered in ARDS patients when compared to controls (from Table S1). As shown in Table 2, 16 pathways with a q < 0.003 were identified. Of those, seven are related to metabolism, four to inflammatory/infection processes, two to addiction processes, one to hormonal alterations, one to cancer and one to Vascular Endothelial Growth Factor (VEGF). This analysis suggests that metabolic alterations/metabolites play an important role in ARDS. We next evaluated if circulating metabolites were altered in ARDS patients.

**Table 2:** Predicted pathways for dysregulated miRNAs. Pathways with a q < 0.003 were included.

Pathways	*P*-value	Q-value	miRNAs
Pantothenate and CoA biosynthesis	6.45E−07	1.51E−04	hsa-miR-16-5p; hsa-miR-375-3p; hsa-miR-345-5p
			hsa-miR-186-5p; hsa-miR-374a-5p; hsa-miR-125b-5p
			hsa-miR-26a-5p; hsa-miR-195-5p; hsa-miR-374b-5p
			hsa-miR-331-3p; hsa-miR-142-3p
Inflammatory bowel disease IBD	1.34E−06	1.51E−04	hsa-miR-181a-5p; hsa-miR-16-5p; hsa-miR-375-3p
			hsa-miR-590-5p; hsa-miR-186-5p; hsa-miR-374a-5p
			hsa-miR-125b-5p; hsa-miR-26a-5p; hsa-miR-195-5p
			hsa-miR-374b-5p; hsa-miR-331-3p; hsa-miR-142-3p
			hsa-miR-139-5p
Nicotinate and nicotinamide metabolism	1.41E−06	1.51E−04	hsa-miR-181a-5p; hsa-miR-16-5p; hsa-miR-375-3p
			hsa-miR-186-5p; hsa-miR-374a-5p; hsa-miR-125b-5p
			hsa-miR-26a-5p; hsa-miR-126-3p; hsa-miR-195-5p
			hsa-miR-374b-5p; hsa-miR-331-3p
Malaria	1.27E−05	8.96E−04	hsa-miR-181a-5p; hsa-miR-16-5p; hsa-miR-375-3p
			hsa-miR-345-5p; hsa-miR-590-5p; hsa-miR-186-5p
			hsa-miR-125b-5p; hsa-miR-26a-5p; hsa-miR-126-3p
			hsa-miR-142-3p; hsa-miR-139-5p
Cocaine addiction	1.40E−05	8.96E−04	hsa-miR-181a-5p; hsa-miR-16-5p; hsa-miR-375-3p
			hsa-miR-590-5p; hsa-miR-186-5p; hsa-miR-374a-5p
			hsa-miR-125b-5p; hsa-miR-126-3p
			hsa-miR-374b-5p; hsa-miR-331-3p; hsa-miR-142-3p
			hsa-miR-139-5p
Rheumatoid arthritis	1.89E−05	0.00101	hsa-miR-181a-5p; hsa-miR-16-5p; hsa-miR-375-3p
			hsa-miR-590-5p; hsa-miR-186-5p; hsa-miR-374a-5p
			hsa-miR-125b-5p; hsa-miR-26a-5p; hsa-miR-126-3p
			hsa-miR-195-5p; hsa-miR-374b-5p; hsa-miR-142-3p
			hsa-miR-139-5p
Fatty acid biosynthesis	3.18E−05	0.001458	hsa-miR-16-5p; hsa-miR-375-3p; hsa-miR-186-5p
			hsa-miR-374a-5p; hsa-miR-26a-5p; hsa-miR-195-5p
			hsa-miR-374b-5p; hsa-miR-331-3p; hsa-miR-142-3p
Phosphonate and phosphinate metabolism	4.62E−05	0.001676	hsa-miR-16-5p; hsa-miR-375-3p; hsa-miR-186-5p
			hsa-miR-374a-5p; hsa-miR-26a-5p; hsa-miR-195-5p
VEGF signaling pathway	4.70E−05	0.001676	hsa-miR-181a-5p; hsa-miR-16-5p; hsa-miR-375-3p
			hsa-miR-345-5p; hsa-miR-186-5p; hsa-miR-374a-5p
			hsa-miR-125b-5p; hsa-miR-26a-5p; hsa-miR-126-3p
			hsa-miR-195-5p; hsa-miR-374b-5p; hsa-miR-331-3p
			hsa-miR-142-3p; hsa-miR-139-5p
Amphetamine addiction	9.90E−05	0.002928	hsa-miR-181a-5p; hsa-miR-16-5p; hsa-miR-375-3p
			hsa-miR-590-5p; hsa-miR-186-5p; hsa-miR-374a-5p
			hsa-miR-125b-5p; hsa-miR-26a-5p; hsa-miR-126-3p
			hsa-miR-374b-5p; hsa-miR-331-3p; hsa-miR-142-3p
			hsa-miR-139-5p
Butanoate metabolism	1.13E−04	0.002928	hsa-miR-16-5p; hsa-miR-375-3p; hsa-miR-186-5p
			hsa-miR-374a-5p; hsa-miR-125b-5p
			hsa-miR-126-3p; hsa-miR-195-5p; hsa-miR-374b-5p
Porphyrin and chlorophyll metabolism	1.16E−04	0.002928	hsa-miR-181a-5p; hsa-miR-16-5p; hsa-miR-375-3p
			hsa-miR-186-5p; hsa-miR-374a-5p
			hsa-miR-125b-5p; hsa-miR-195-5p
			hsa-miR-374b-5p; hsa-miR-331-3p; hsa-miR-142-3p
Parathyroid hormone synthesis, secretion and action	1.21E−04	0.002928	hsa-miR-181a-5p; hsa-miR-16-5p; hsa-miR-375-3p
			hsa-miR-345-5p; hsa-miR-590-5p; hsa-miR-186-5p
			hsa-miR-374a-5p; hsa-miR-125b-5p
			hsa-miR-26a-5p; hsa-miR-126-3p; hsa-miR-195-5p
			hsa-miR-374b-5p; hsa-miR-331-3p; hsa-miR-142-3p
			hsa-miR-139-5p
Vitamin B6 metabolism	1.33E−04	0.002928	hsa-miR-16-5p; hsa-miR-186-5p; hsa-miR-125b-5p
			hsa-miR-26a-5p; hsa-miR-195-5p; hsa-miR-331-3p
Endometrial cancer	1.41E−04	0.002928	hsa-miR-181a-5p; hsa-miR-16-5p; hsa-miR-375-3p
			hsa-miR-345-5p; hsa-miR-590-5p; hsa-miR-186-5p
			hsa-miR-374a-5p; hsa-miR-125b-5p
			hsa-miR-26a-5p; hsa-miR-126-3p; hsa-miR-195-5p
			hsa-miR-374b-5p; hsa-miR-331-3p; hsa-miR-142-3p
			hsa-miR-139-5p
Inflammatory mediator regulation of TRP channels	1.46E−04	0.002928	hsa-miR-181a-5p; hsa-miR-16-5p; hsa-miR-375-3p
			hsa-miR-345-5p; hsa-miR-186-5p; hsa-miR-374a-5p
			hsa-miR-125b-5p; hsa-miR-26a-5p; hsa-miR-126-3p
			hsa-miR-195-5p; hsa-miR-374b-5p; hsa-miR-331-3p
			hsa-miR-142-3p; hsa-miR-139-5p

### Evaluation of circulating metabolites in a subset of ARDS and control patients

**Figure 4:**
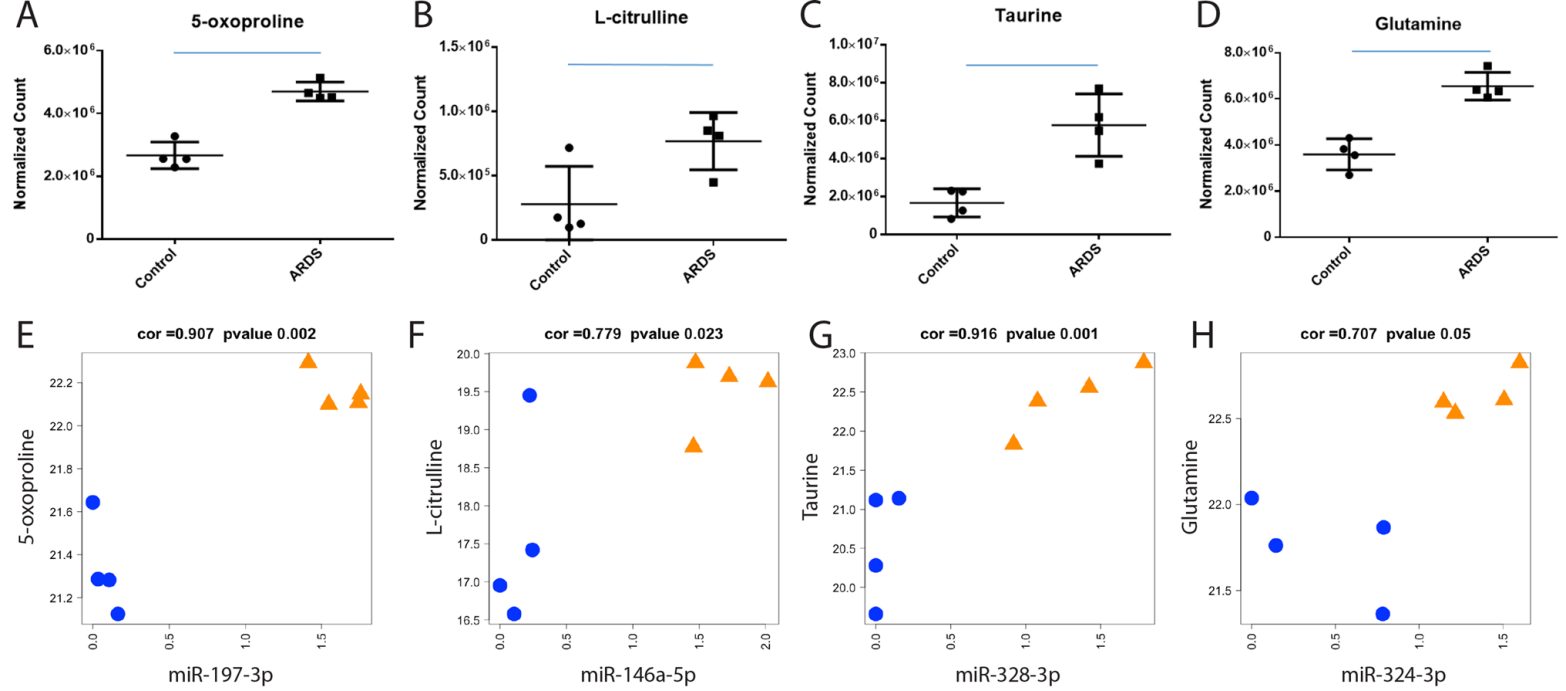
Correlation of circulating metabolites with circulating miRNAs (n = 4 ARDS, n = 4 control). (**A**–**D**) Normalized count detection for 5-oxoproline, L-citrulline, Taurine, and glutamine respectively. Statistical significance determined by *t*-test, indicated by blue bars. (**E**–**H**) miRNA and metabolite abundance for pairs with the highest correlation. ARDS-acute respiratory distress syndrome, CTL-control.

In addition to miRNAs, circulating metabolites have received recent attention as a powerful biomarker for human disease^12^. Therefore, we selected a subset of four ARDS and four control patients that grouped together by unsupervised hierarchical clustering to evaluate the relative quantity of circulating metabolites (Fig. 1E, ARDS group far left, control group far right). Seven metabolites increased in ARDS patients relative to control, defined by an absolute fold change greater than 1.5 and *p* < 0.05 (Table S4). 5-oxoproline, L-citrulline, glutamine, and taurine were differentially abundant and have previously described relationships with ARDS (Fig. 4A–D). The changes in metabolites were then tested to determine if the differential abundance correlated with alterations in miRNA levels. Metabolite abundance strongly correlated (absolute r > 0.7, *p* < 0.05) with select miRNA abundance (Fig. S2). Of the differentially expressed metabolites, the highest correlation coefficients were found for 5-oxoproline-miRNA-197-3p (r = 0.90; *p* = 2.49E−3), L-citrulline-miRNA-146a-5p (r = 0.80, *p* = 1.68E−2), Taurine-miRNA-328-3p (r = 0.90, *p* = 2.43E − 3), and Glutamine-miRNA-342-3p (r = 0.86, *p* = 6.04E−3) (Fig. 4E–H and Fig. S2). The significantly altered KEGG pathways related to these metabolites are shown in Table S.

## Discussion

ARDS is a highly heterogeneous syndrome of severe respiratory failure resulting from diverse etiologies. Accumulating evidence indicates that sub-phenotypes of ARDS may respond differently to therapeutic interventions^5^. These findings suggest that developing biomarkers to more accurately identify ARDS or sub-phenotypes of ARDS may be of great clinical utility. In addition, while evidence is emerging that circulating miRNAs may be useful biomarkers in adult ARDS patients, the role of circulating miRNAs in pediatric ARDS remains largely unexplored. We demonstrate here that circulating miRNAs are detectable in critically ill children, that circulating miRNA expression patterns differ between patients with ARDS and control patients, and that circulating miRNA patterns differ depending on the underlying etiology of ARDS.

Using RFC and hierarchical clustering, we found that ARDS and control patients were accurately differentiated by a three-miRNA biomarker panel of miRNA-345-5p, -375-3p, and -126-3p (Fig. 1). This is consistent with other studies that have used 1–4 miRNAs to assess ARDS risk in sepsis^25^, severe community acquired pneumonia^26^, and critical illness^27^. Intriguingly, miRNA-126 was identified previously as a biomarker differentiating adult patients who did or did not progress to ARDS in the setting of severe community acquired pneumonia^26^. And importantly, miR-126-3p was recently defined as a biomarker of mortality in pediatric ARDS patients^28^. This is the first time miRNAs-345-5p and -375-3p have been identified as biomarkers for ARDS, though they have previously been implicated as biomarkers for pediatric diabetes^29^. The fact that these differences were seen early in the course of ARDS and in comparison to a critically ill and mechanically ventilated control group raises the possibility that miRNA profiling could be of use in identifying ARDS patients at an early time-point in their course and inform therapeutic interventions.

The most common etiologies of ARDS in children are respiratory infections. We observed that the specificity of patient grouping increased substantially when the analysis was limited to patients with a documented bacterial respiratory infection (Fig. 2). Among patients with bacterial LRTI, the three miRNAs that best discriminated ARDS from control patients (-590-5p, -324-3p, and -486-3p) have been described as biomarkers in the settings of COVID-19 infection and lung cancer^30,31^. Notably, miRNAs-486-3p has also been identified as a biomarker of sepsis, a common cause of ARDS^32^. This is the first time these three miRNAs have been identified as circulating biomarkers of ARDS in bacterially infected patients. The ability of miRNAs to distinguish ARDS and control patients in the bacterial-infection subset indicates that stratification of patients could further enhance the prognostic value of three-miRNA panels.

Given that RFC differentiated ARDS and control patients more accurately when subset by bacterial infection, we next sought to classify infection type using circulating miRNAs. RFC and hierarchical clustering using the top three miRNAs (miRNA-150-5p, -192-5p, -548a-3p) differentiated patients by infection type (Fig. 3). Thus, circulating miRNAs could be used to support patient stratification and application of the sub-grouped 3-miRNA panels. The size of our viral cohort precluded differentiation of ARDS and control patients in this subgroup, though miRNAs have been identified as dysregulated in response to viral infection previously^33,34^. We speculate that a larger cohort of patients with viral infection would inform an analysis to improve patient stratification.

Pathway analysis suggested miRNAs altered in ARDS patients were overwhelmingly predictive of altered metabolic pathways. In addition, inflammatory/infectious processes, VEGF signaling, drug treatment, cancer and hormonal imbalances were also predicted to be affect by these miRNAs. As ARDS is an inflammatory process, inflammation/infection pathways were expected, as were pathways related to possible drug treatments (cocaine and amphetamine addiction). VEGF induces vascular permeability, and anti-VEGF treatment attenuated lung injury in a mouse model of ARDS^35^.

Metabolic regulation is central to inflammatory processes, which may explain the large number of predicted altered metabolic pathways^36^. As not much is known on levels of circulating metabolites in ARDS patients, we investigated, in a subset of patients, if circulating metabolites were altered and their relationship to circulating miRNAs. Measurement of circulating metabolites in four control and four ARDS patients identified seven differentially abundant metabolites (Table S4). 5-oxoproline^37^ and taurine^11^ have been described as biomarkers of human lung injury consistent with our data. L-citrulline^38^ and Taurine^39^ have also been found to be upregulated in preclinical models of lung injury. Conversely, L-citrulline and 5-oxoproline have been found to be lower in patients with lung injury in the settings of sepsis and tuberculosis respectively^40,41^. It appears that the dysregulation of these metabolites is dependent on the primary condition leading to ARDS. Functionally, L-citrulline^42^, Taurine^43^, and Glutamine^44,45^ have been shown to ameliorate lung injury. This suggests that these metabolites are upregulated endogenously as a response to injury. Circulating metabolite abundance also correlated with circulating miRNA expression (Fig. 4). It is unknown if there is a coordinated response between the release of miRNAs and metabolites in the circulation, or if disease-specific processes coordinately affect various organs resulting in the release of these circulating factors from different sources. The strong correlation between miRNAs and metabolites suggests that metabolites could be used as an alternative or supplementary biomarker to enhance discrimination of patients. A larger cohort of samples is needed to determine if combined metabolomic and miRNomic can more accurately identify patients than any single circulating factor.

There are limitations to this study. First, a greater sample size is necessary to validate these initial findings and determine the predictive value of the miRNAs identified in this study. Similar expansion of the metabolomics cohort could enable combined predictive modeling of ARDS using metabolites and miRNAs, an approach that has been applied to other diseases^46^. Second, quantification of plasma miRNAs combines the contents of the extracellular fluid, exosomes, and other non-cellular sources. We did not identify the source of these miRNAs, or their origin. Exosome isolation requires a much higher volume of specimen than what our methodology requires. Since these are pediatric patients, sample collection volume was limited by weight. Also, we applied a modified Berlin ARDS definition rather than the broader PALICC definition, and thus these findings may not be generalizable to patients meeting only the PALICC ARDS definition. Lastly, although samples were collected relatively early in their course for all patients, variation in the timing of sample collection between patients is also a limitation, especially as we were not able in this study to assess trajectories of miRNA expression with time. To better define the utility of miRNA expression patterns for diagnosing PARDS, a larger prospective study with stricter sample timing and serial sampling will be necessary.

## Conclusion

In summary, we have demonstrated that circulating plasma miRNA profiles may not only be able to identify pediatric patients with ARDS early in the course of their illness but also may be able to provide information about the underlying infectious etiology. Further work in a larger cohort will be necessary to validate these findings and further explore the potential utility of miRNA profiling in a systems biology approach to ARDS diagnosis and treatment in children.

## Supplementary Information

Supplementary Information.

## Data Availability

The datasets generated during and/or analyzed during the current study are available from the corresponding author on reasonable request.

## Abbreviations

ARDS: Acute respiratory distress syndrome
PARDS: Pediatric acute respiratory distress syndrome
miRNAs: MicroRNAs
PICU: Pediatric Intensive Care Unit
PELOD: Pediatric Logistic Organ Dysfunction
RFC: Random forest classification
ROC: Receive operating curve
AUC: Area under the curve
LRTI: Lower respiratory tract infections
