# Supplementary material for: Plasma microRNA and metabolic changes associated with pediatric acute respiratory distress syndrome: a prospective cohort study
Source: Sci Rep. 2022 Aug 26;12:14560. doi: 10.1038/s41598-022-15476-0 (PMC9418138; doi:10.1038/s41598-022-15476-0)

**Supplemental Data**

Table S1: Differentially expressed miRNAs acute respiratory distress vs control. Significance tested by Wilcoxon's t-test (n=21 acute respiratory distress, n=14 control).

| miRNA | Fold Change | P-value |
|---|---|---|
| miR-181a-5p | 1.88 | 4.11E-03 |
| miR-16-5p | 1.91 | 6.97E-03 |
| miR-375 | 1.62 | 8.47E-03 |
| miR-345-5p | 1.29 | 8.68E-03 |
| miR-590-5p | 1.45 | 1.33E-02 |
| miR-186-5p | 1.28 | 1.40E-02 |
| miR-374a-5p | 1.82 | 1.60E-02 |
| miR-125b-5p | 1.57 | 1.80E-02 |
| miR-26a-5p | 1.80 | 2.46E-02 |
| miR-126-3p | 1.78 | 2.60E-02 |
| miR-195-5p | 1.49 | 3.12E-02 |
| miR-374b-5p | 1.87 | 3.24E-02 |
| miR-331-3p | 1.48 | 4.16E-02 |
| miR-142-3p | 1.75 | 4.41E-02 |
| miR-139-5p | 1.59 | 4.88E-02 |

Table S2: Differentially expressed miRNAs in patients with bacterial LRTI, ARDS vs control. Significance tested by Wilcoxon's t-test (n=11 ARDS, n=8 control). ARDS-Acute respiratory distress syndrome, LRTI-lower respiratory tract infection.

| miRNA | Fold Change | P-value |
|---|---|---|
| miR-324-3p | 1.47 | 2.54E-03 |
| miR-186-5p | 1.38 | 1.57E-02 |
| miR-590-5p | 1.59 | 1.86E-02 |
| miR-200c-3p | 1.88 | 1.99E-02 |
| miR-363-3p | 1.66 | 2.29E-02 |
| miR-374b-5p | 2.41 | 3.17E-02 |
| miR-20b-5p | 1.91 | 3.28E-02 |
| miR-223-3p | 1.71 | 3.28E-02 |
| miR-486-3p | 1.55 | 3.56E-02 |
| miR-195-5p | 1.85 | 3.89E-02 |
| miR-331-3p | 1.80 | 3.89E-02 |
| miR-26a-5p | 2.02 | 3.99E-02 |
| miR-16-5p | 2.11 | 4.09E-02 |
| miR-376a-3p | 1.88 | 4.27E-02 |
| miR-181a-5p | 1.89 | 4.65E-02 |
| miR-374a-5p | 2.10 | 4.74E-02 |

Table S3: Differentially expressed miRNAs in acute respiratory distress patients, bacterial and viral lower respiratory tract infection vs viral infection alone. Significance tested by Wilcoxon's t-test (n=10 bacterial, n=9 viral).

| miRNA | Fold Change | P-value |
|---|---|---|
| miR-192-5p | 1.68 | 7.62E-03 |
| miR-532-3p | 1.88 | 1.01E-02 |
| miR-486-3p | 1.78 | 1.51E-02 |
| miR-150-5p | 1.98 | 1.72E-02 |
| miR-let-7b-5p | 2.03 | 2.20E-02 |
| miR-548a-3p | -1.54 | 2.47E-02 |
| miR-140-3p | 1.39 | 2.79E-02 |
| miR-215-5p | 1.65 | 2.79E-02 |
| miR-17-5p | 1.40 | 3.50E-02 |
| miR-324-3p | 1.32 | 3.50E-02 |
| miR-195-5p | 1.63 | 4.35E-02 |
| miR-486-5p | 1.57 | 4.99E-02 |

Table S4: Differentially abundant metabolites acute respiratory distress vs control. Significance tested by t-test (n=4).

| Metabolite | Median Abundance Control | Median Abundance ARDS | Fold Change | P- value |
|---|---|---|---|---|
| 5-Oxoproline | 2.55E+06 | 4.59E+06 | 1.80E+00 | 2.31E-04 |
| Glutamine | 3.69E+06 | 6.36E+06 | 1.73E+00 | 5.98E-04 |
| Taurine | 1.77E+06 | 5.83E+06 | 3.29E+00 | 3.89E-03 |
| N-Succinyl-L-citrulline | 2.58E+05 | 4.81E+05 | 1.86E+00 | 1.91E-02 |
| Glycerone phosphate | 3.93E+07 | 4.75E+07 | 1.21E+00 | 2.65E-02 |
| Leucine | 2.95E+07 | 4.78E+07 | 1.62E+00 | 3.08E-02 |
| L-Citrulline | 1.51E+05 | 8.32E+05 | 5.50E+00 | 3.78E-02 |
| Cysteine | 1.14E+05 | 1.98E+05 | 1.74E+00 | 4.26E-02 |
| UDP | 2.34E+06 | 1.75E+06 | 7.47E-01 | 4.93E-02 |

Table S5: Significantly predicted pathways based on dysregulated miRNAs that include altered metabolites

| Metabolites | Pathways | P-value | Q-value | miRNAs |
|---|---|---|---|---|
| 5-oxoproline | Glutathione metabolism | 9.40E-03 | 2.05E-02 | hsa-miR-181a-5p; hsa-miR-16-5p; hsa-miR-186-5p; hsa-miR-125b-5p; hsa-miR-26a-5p; hsa-miR-195-5p; hsa-miR-331-3p; hsa-miR-142-3p |
| l-citrulline | Arginine biosynthesis | 2.48E-02 | 4.24E-02 | hsa-miR-181a-5p; hsa-miR-16-5p; hsa-miR-186-5p; hsa-miR-26a-5p; hsa-miR-142-3p |
| Taurine | Primary bile acid biosynthesis | 3.72E-03 | 1.12E-02 | hsa-miR-16-5p; hsa-miR-186-5p; hsa-miR-26a-5p; hsa-miR-142-3p |
| Taurine | ABC transporters | 2.23E-04 | 3.15E-03 | hsa-miR-181a-5p; hsa-miR-16-5p; hsa-miR-345-5p; hsa-miR-186-5p; hsa-miR-374a-5p; hsa-miR-125b-5p; hsa-miR-26a-5p; hsa-miR-195-5p; hsa-miR-374b-5p; hsa-miR-142-3p |
| Taurine | Neuroactive ligand-receptor interaction | 6.70E-03 | 1.65E-02 | hsa-miR-181a-5p; hsa-miR-16-5p; hsa-miR-375-3p; hsa-miR-345-5p; hsa-miR-186-5p; hsa-miR-374a-5p; hsa-miR-125b-5p; hsa-miR-26a-5p; hsa-miR-126-3p; hsa-miR-195-5p; hsa-miR-374b-5p; hsa-miR-331-3p; hsa-miR-142-3p; hsa-miR-139-5p |
| Glutamine | Arginine biosynthesis | 2.48E-02 | 4.24E-02 | hsa-miR-181a-5p; hsa-miR-16-5p; hsa-miR-186-5p; hsa-miR-26a-5p; hsa-miR-142-3p |
| Glutamine | Pyrimidine metabolism | 1.00E-02 | 2.14E-02 | hsa-miR-16-5p; hsa-miR-375-3p; hsa-miR-186-5p; hsa-miR-125b-5p; hsa-miR-26a-5p; hsa-miR-195-5p; hsa-miR-374b-5p; hsa-miR-331-3p; hsa-miR-142-3p |
| Glutamine | Vitamin B6 metabolism | 1.33E-04 | 2.93E-03 | hsa-miR-16-5p; hsa-miR-186-5p; hsa-miR-125b-5p; hsa-miR-26a-5p; hsa-miR-195-5p; hsa-miR-331-3p |
| Glutamine | Nitrogen metabolism | 1.18E-03 | 6.04E-03 | hsa-miR-16-5p; hsa-miR-186-5p; hsa-miR-374a-5p; hsa-miR-26a-5p; hsa-miR-195-5p; hsa-miR-374b-5p; hsa-miR-142-3p |
| Glutamine | ABC transporters | 2.23E-04 | 3.15E-03 | hsa-miR-181a-5p; hsa-miR-16-5p; hsa-miR-345-5p; hsa-miR-186-5p; hsa-miR-374a-5p; hsa-miR-125b-5p; hsa-miR-26a-5p; hsa-miR-195-5p; hsa-miR-374b-5p; hsa-miR-142-3p |
| Glutamine | Glutamatergic synapse | 2.30E-03 | 8.68E-03 | hsa-miR-181a-5p; hsa-miR-16-5p; hsa-miR-375-3p; hsa-miR-345-5p; hsa-miR-186-5p; hsa-miR-374a-5p; hsa-miR-125b-5p; hsa-miR-26a-5p; hsa-miR-126-3p; hsa-miR-195-5p; hsa-miR-374b-5p; hsa-miR-331-3p; hsa-miR-142-3p |
| Glutamine | GABAergic synapse | 3.19E-03 | 1.03E-02 | hsa-miR-181a-5p; hsa-miR-16-5p; hsa-miR-375-3p; hsa-miR-186-5p; hsa-miR-374a-5p; hsa-miR-125b-5p; hsa-miR-26a-5p; hsa-miR-195-5p; hsa-miR-374b-5p; hsa-miR-331-3p; hsa-miR-142-3p; hsa-miR-139-5p |

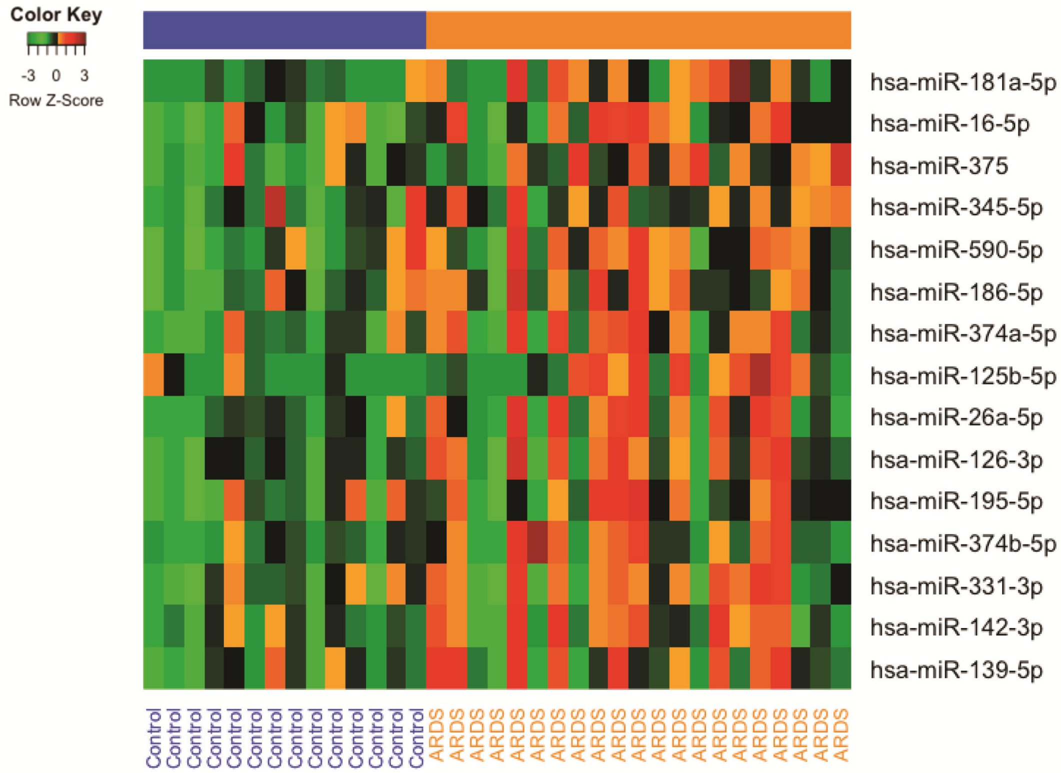


Fig S1: Differentially expressed microRNAs in ARDS vs control samples quantified by microarray. Fifteen microRNAs were differentially expressed ($p < 0.05$) by Wilcoxon rank sum test, n=21 ARDS, n=14 control. ARDS-Acute Respiratory Distress Syndrome.

Fig S2: Circos plot of correlations with a $p < 0.05$ and r value > 0.7 between miRNAs and metabolites.

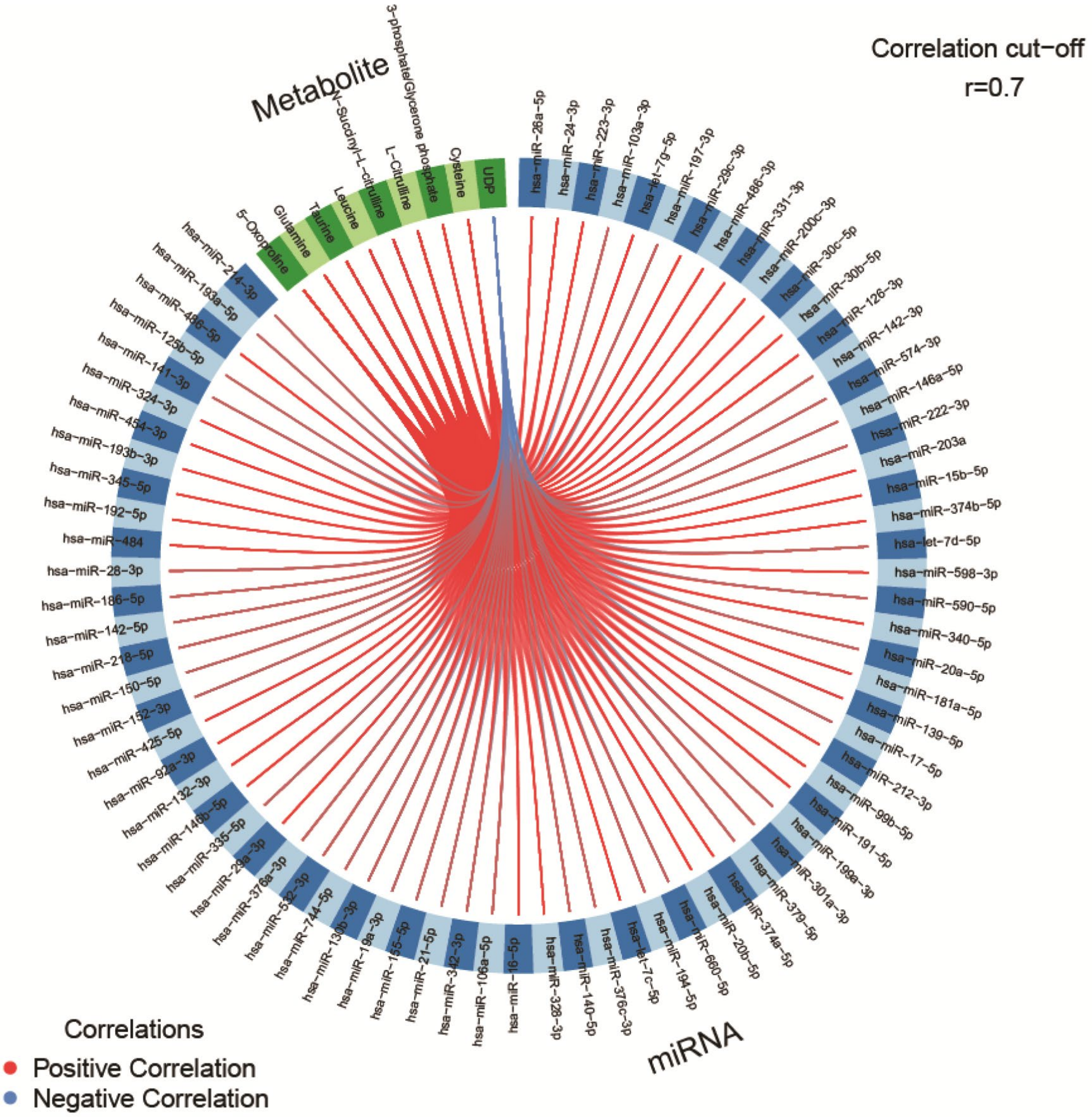

Supplement: Supplementary file 1 — Supplementary Information. [file 41598_2022_15476_MOESM1_ESM.pdf]
